# Editorial: Recent advances in multimodal artificial intelligence for disease diagnosis, prognosis, and prevention

**DOI:** 10.3389/fradi.2023.1349830

**Published:** 2024-01-10

**Authors:** Hazrat Ali, Zubair Shah, Tanvir Alam, Priyantha Wijayatunga, Eyad Elyan

**Affiliations:** ^1^ College of Science and Engineering, Hamad Bin Khalifa University, Doha, Qatar; ^2^ Department of Statistics Umeå University, Umeå, Sweden; ^3^ School of Computing Science and Digital Media, Robert Gordon University, Aberdeen, United Kingdom

**Keywords:** electronic health records, healthcare, medical imaging, radiology, multimodal artificial intelligence, vision transformers

**Editorial on the Research Topic** Recent advances in multimodal artificial intelligence for disease diagnosis, prognosis, and prevention

## Introduction

Artificial Intelligence (AI) has gained huge attention in computer-aided decision-making in the healthcare domain. Many novel AI methods have been developed for disease diagnosis and prognosis which may support in the prevention of disease. Most diseases can be cured early and managed better if timely diagnosis is made. The AI models can aid clinical diagnosis; thus, they make the processes more efficient by reducing the workload of physicians, nurses, radiologists, and others. However, the majority of AI methods rely on the use of single-modality data. For example, brain tumor detection uses brain MRI, skin lesion detection uses skin pathology images, and lung cancer detection uses lung CT or x-ray imaging (1). Single-modality AI models lack the much-needed integration of complex features available from different modality data, such as electronic health records (EHR), unstructured clinical notes, and different medical imaging modalities– otherwise form the backbone of clinical decision-making.

Many recent studies have demonstrated that the use of multimodal data tends to enhance the predictive performance of the AI models in medical imaging, e.g., leveraging diverse set of features from different modalities (2). Hence, multimodal AI models, along with early/late data/inference fusion approaches, can utilize complex features from data efficiently, thus resulting in better decisions. But, combining data from different modalities is not straightforward as the data differ in dimensionality, modality, as well as availability. Exploring new methods to combine different data types often leads to new protocols and strategies for multimodal data collection, cleaning, pre-processing, and integration. Hence, many practical challenges exist in achieving the goal of training multimodal AI models for medical data for accurate prediction, explanation, etc. Therefore, there is a big need to cover recent advancements and novel methods and techniques in AI for multimodal healthcare data or multimodal AI in healthcare.

This Research Topic comprises contributions from experienced researchers and scientist in artificial intelligence, radiology, and healthcare, covering recent works on (i) improving explainability of deep learning-based AI methods in radiology, (ii) new methods of adversarial counterfactual augmentation to achieve generalization of AI model for Alzheimer’s Disease classification, (iii) a comprehensive review of vision transformer-based methods in improving skin cancer diagnosis, (iv) a scoping review of the recent multimodal AI methods that combine medical images and electronic health record data for the diagnosis and prognosis of liver cancer (hepatocellular carcinoma).

The first study addresses the inherent challenge of imparting explicability to AI models in radiology, with the ultimate goal of fostering trust among clinicians Watanabe et al. The study scrutinizes the efficacy of the U-Net model in classifying congestive heart failure and pneumonia diseases using chest radiographs. Notably, the investigation incorporates the spatial information of radiologists’ eye-gaze coordinates, illustrating that the integration of multimodal data facilitates the development of AI models with enhanced explicability and overall classification performance. Significantly, the study demonstrates the efficacy of integrating heatmap generators and eye-gaze information during AI model training, aligning with the interpretative approach of radiologists in the analysis of chest radiographs.

The second study delves into the efficacy of augmentation techniques in the training of deep learning models Xia et al. Principally, the study introduces an adversarial counterfactual approach for data augmentation grounded in the identification of optimally synthesized images conducive to enhancing performance in downstream tasks. The counterfactual approach is an instance of sematic augmentation techniques where new data/examples are created through changing sematic information such as age of the patient, etc. In contrast to traditional methods such as augmenting synthetic data through reinforcement learning using certain policies, this study focuses on finding the weakness of the downstream task and forcing it to overcome the respective weaknesses. Validation of this novel method is undertaken in the context of Alzheimer's Disease, where brain images are synthesized with age as a conditional factor.

The third study furnishes an exhaustive review regarding the utilization of vision transformers in the context of skin cancer detection Khan et al. The identification of skin lesions poses a formidable challenge, typically addressed through the utilization of dermoscopy images for skin cancer detection. The review underscores a notable surge in the exploration of vision transformers for this purpose. However, pervasive challenges, including the irregular shapes of skin lesions and inherent visual ambiguities, present formidable obstacles in advancing AI methodologies for skin cancer detection, thereby constraining the reliability of such approaches. The review imparts novel perspectives to readers regarding the precise demarcation of lesion boundaries and the detection of melanoma.

In the fourth study, a scoping review is presented, examining multimodal artificial intelligence (AI) approaches for liver cancer, a leading global cause of cancer-related mortality Siam et al. The focus of the review lies in initiatives that integrate medical imaging with electronic health record data for the purpose of developing AI methods tailored to hepatocellular carcinoma diagnosis. Computed tomography and magnetic resonance imaging emerge as the most commonly utilized imaging modalities, while clinical factors such as age, gender, alpha-fetoprotein, and albumin stand out as prevalent contributors to liver cancer identification.

Overall, all the studies covered under this Research Topic suggest that the advancement of multimodal AI methodologies in healthcare holds significant potential, offering insights that might otherwise remain obscured. We believe the incorporation of multimodal AI models in healthcare setup is warranted in near future.
